# Metabolic and immune dysfunctions in post-traumatic stress disorder: what can we learn from animal models?

**DOI:** 10.17179/excli2023-6391

**Published:** 2023-09-04

**Authors:** Oleh Lushchak, Marco Orru, Olha Strilbytska, Vladyslav Berezovskyi, Andriy Cherkas, Kenneth B. Storey, Maria Bayliak

**Affiliations:** 1 Precarpathian National University, Ivano-Frankivsk, Ukraine; 2 Research and Development University, Ivano-Frankivsk, Ukraine; 3 Carleton University, Ottawa, Canada

**Keywords:** post-traumatic stress disorder, metabolism, immunity

## Abstract

Highly stressful experiences such as terrorist attacks, domestic and sexual violence may lead to persistent pathological symptoms such as those seen in posttraumatic stress disorder (PTSD). There is growing evidence of multiple metabolic and immune disorders underlying the etiology and maintenance of PTSD. However, changes in the functioning of various systems and organs associated with PTSD are not well understood. Studies of reliable animal models is one of the effective scientific tools that can be used to gain insight into the role of metabolism and immunity in the comorbidity associated with PTSD. Since much progress has been made using animal models to understand mechanisms of PTSD, we summarized metabolic and immune dysfunction in mice and humans to compare certain outcomes associated with PTSD. The systemic effects of PTSD include chronic activation of the sympathetic nervous system (psycho-emotional stress), that leads to impairment of the function of the immune system, increased release of stress hormones, and metabolic changes. We discuss PTSD as a multisystem disease with its neurological, immunological, and metabolic components.

## Introduction

Post-traumatic stress disorder (PTSD) is a psychiatric condition that can occur in people who have experienced traumatic, shocking, or dangerous events. Past research on PTSD mainly focused on war veterans who personally experienced or witnessed traumatic events and is linked to psychological functioning impairment across various domains, including family, marriage, employment, and education. However, the increase in challenges in our society have brought PTSD to the center of attention not only because of combat related trauma. Indeed, recent studies showed that people and families living in generally safe communities are also being exposed increasingly to factors such as terrorist attacks, domestic and sexual violence that may lead to PTSD (Dworkin et al., 2017[17]). 

The prevalence of PTSD across the world is estimated to be about 4-6 % of the total population but reaching roughly 8 % of adults in the United States (Atwoli et al., 2015[6]). In addition, the COVID-19 pandemic caused massive distress in large population groups, primarily COVID-19 patients themselves and healthcare workers on the frontline (medical doctors, nurses, paramedics, etc.) (Li et al., 2023[42]; Horesh and Brown, 2020[25]). There is also increasing evidence that extended lockdowns are often accompanied by stress related to loss of income (economic crisis) that can contribute to long-term deterioration of mental health in vulnerable populations including children, young individuals, women, etc. (American Psychiatric Association, 2013[3]). Altogether that makes PTSD a serious challenge to mental health on a global scale (Horesh and Brown, 2020[25]). Despite the wide spread of prevalence in PTSD, the general awareness of this serious disorder remains rather low and diagnostic and treatment options are often of limited availability or efficacy. The issue of the substantial rise of PTSD rates is of growing importance as this disorder is a serious risk factor for alcohol and drug abuse (Alexander, 2012[2]), depression, suicide, and functional dysregulation of internal organs, particularly of cardiovascular (Kar, 2011[33]), endocrine (Tawa and Murphy, 2013[87]), and immune systems (Difede et al., 2014[15]; Kamo et al., 2016[32]). PTSD can also lead to social disadaptation in the family and at work (Hoskins et al., 2015[27]). The severity of PTSD symptoms is also associated with accelerated cognitive decline (Prieto et al., 2023[74]). This makes PTSD a very important social and public health problem that causes significant economic damage, since most individuals with PTSD are young and of working age.

Being exposed to traumatic events is a required risk factor for the development of PTSD but not the only factor (Flory et al., 2015[20]; Dursa et al., 2014[16]). In addition to traditional symptoms, individuals with PTSD, typically have one or more co-morbidity such as obesity, type 2 diabetes (T2DM), metabolic syndrome, and increased rates of cardiovascular diseases and, as a consequence, early mortality (Mellon et al., 2018[57]; Levine et al., 2014[40]; Blessing et al., 2017[10]; Rosenbaum et al., 2015[78]). These systemic metabolic dysregulations or simply changes in metabolism because of traumatic experiences, along with lifestyle-related factors, have been proposed as possible contributors to somatic disease risk in PTSD (Michopoulos et al., 2016[61]).

Dysregulation of the immune system may contribute towards central nervous system tissue damage and exacerbation of fear memories following trauma (Hori and Kim, 2019[26]). Although some studies have shown that results of immune alterations associated with PTSD are somehow contradictory, the presence of inflammatory states in subjects with PTSD is real. Although the high co-morbidity between inflammatory processes, metabolic disorders and PTSD suggest that inflammatory and metabolic changes increase the risk for systemic and psychiatric dysregulation, at the present time, the precise mechanisms of PTSD pathogenesis remain unclear (Lindquist et al., 2014[45]; McFarlane et al., 2017[54]).

The current review summarizes evidence about metabolic and inflammatory dysregulations in animal models of PTSD and illustrates some factors that may play a role in the psychopathological burden of PTSD. We also offer a comprehensive assessment of studies in discovering pathways or networks with a focus on immune mechanisms and metabolic dysfunction that are involved after trauma. Specifically, emphasis is placed on the importance of animal studies of PTSD for identifying potential targets for novel pharmacotherapies, and screening drugs for their potential use as PTSD treatments in humans.

## Metabolic Dysfunction in Individuals with PTSD

PTSD has previously been linked to an increased risk of a wide range of health problems including obesity, metabolic syndrome, heart disease, autoimmune disorders, and type 2 diabetes (T2DM) (Blessing et al., 2017[10]; Michopoulos et al., 2016[61]; Masodkar et al., 2016[53]; Rosenbaum et al., 2015[78]). Metabolic dysregulation or primary changes in human metabolism because of traumatic experiences correspond to high co-morbidity between metabolic disorders and PTSD (Mellon et al., 2018[57]; Lihua et al., 2020[44]). It was recently discussed that mitochondria modulate the effects of psychological stress on metabolic perturbations (Lushchak et al., 2023[47]).

Metabolic syndrome (MS) is typically characterized by the presence of increased abdominal fat mass, hyperglycemia, elevated blood pressure, increased levels of triglycerides and decreased levels of high-density lipoprotein (HDL) (Michopoulos et al., 2016[61]). Veterans with combat-related PTSD as well as veterans with PTSD comorbid with major depressive disorder showed significantly higher concentrations of cholesterol, triglycerides, low-density lipoprotein cholesterol (LDL-C) (Karlović et al., 2004[34]). However, patients with PTSD caused by myocardial infarction (MI) had lower HDL-C than those with no PTSD (von Känel et al., 2010[97]). It was demonstrated altered metabolites level involved in glucose metabolism, energy utilization and lipid metabolism in male combat trauma-exposed veterans from the Iraq and Afghanistan conflicts with PTSD (Mellon et al., 2019[56]). The presence of metabolic disorders is highly predictive of future cardiovascular events and could contribute to the increase in co-morbidity and mortality of PTSD (Rosenbaum et al., 2015[78]; Wolf and Schnurr, 2016[106]). As shown in a study of combat-related trauma in men, the prevalence of MS was significantly higher in individuals with PTSD (18.8 versus 1.3 %) (Blessing et al., 2017[10]). In a military cohort study started in 2001 and involving more than 77,000 military service members, binge eating disorder was investigated and linked to weight gain related to PTSD (Mitchell et al., 2016[64]). At the end of the study some of the results showed that the percentage of veterans and military personnel that were considered overweight in 2013 was almost 8 % compared to 2001 when it was only 1.6 % (Mitchell et al., 2016[64]). An increased rate of obesity, especially in the military, has huge implications for long-term healthcare because it significantly elevates the likelihood of hypertension, diabetes, and sleep apnea compared to individuals of normal weight (Kubzansky et al., 2014[38]).

However, PTSD symptoms connected to metabolic dysregulation were not found only in war veterans or service men. In fact, an interesting link between obesity and PTSD was also shown in a study published in 2014 in JAMA Psychiatry. During this study, nurses who had a healthy weight when they joined the study but eventually went on to develop symptoms of PTSD, were more likely to gain weight than women who experienced a traumatic event but remained free of PTSD symptoms (Kubzansky et al., 2014[38]). The data showed that normal-weight nurses with four or more PTSD symptoms over a month or longer were 36 % more likely to become overweight or obese compared to those who had experienced trauma but had no symptoms of PTSD (18 %). Among these nurses, common symptoms of PTSD included re-experiencing the traumatic event, feeling under threat, social avoidance, and numbness. Moreover, when these data were adjusted for depression, which has also been proposed as a major risk factor for obesity, the risk of co-morbidity between PTSD and obesity was even more evident. Another recent meta-analysis study found an association between PTSD and increased body mass index (BMI) suggesting that women may have a higher risk of developing weight problems following PTSD onset. Out of this analysis, in 17 of the 30 studies chosen that included 191,948 people with PTSD and 418,690 people without the disorder, women who had PTSD showed significantly higher BMI values than the healthy controls (Suliman et al., 2016[85]). This evidence was further supported by a recent study that investigated genetic correlations of PTSD and MS and found significant genetic overlap between the two (Misganaw et al., 2022[63]). Hence, genetics may play a part in the increased occurrence of MS in individuals with PTSD.

**Figure 1:**
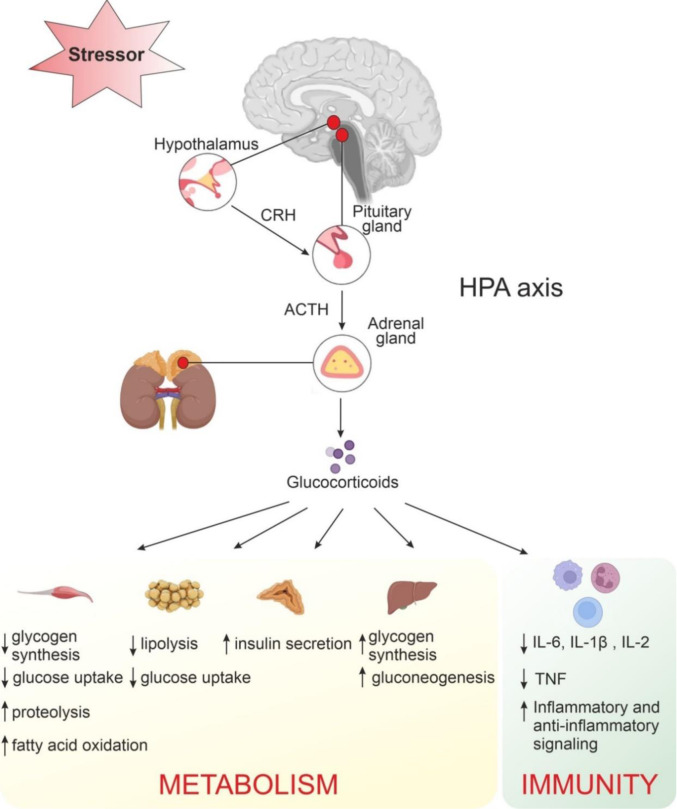
An overview of the neurological, metabolic and immunity imbalances in patients with PTSD. The hypothalamic-pituitary-adrenal (HPA) axis is the primary neuroendocrine pathway involved in stress response via release of secretions. Glucocorticoids appear to regulate various metabolic and immune processes. CRH, corticotropin-releasing hormone; ACTH, adrenocorticotrophic hormone; TNF, tumor necrosis factor; IL-6, interleukin-6; IL-1β, interleukin-1β; IL-2, interleukin-2.

Although there is a huge amount of literature linking PTSD to MS and obesity, unfortunately, the biological pathway that leads people with PTSD to develop these metabolic disturbances is still unknown. Furthermore, there is no clear strategy currently for treating or preventing PTSD associated outcomes. In a review published by Aaseth and colleagues (2019[1]), the authors focused their attention on dysregulation of function of the hypothalamic-pituitary-adrenal axis (HPA) and the sympathetic nervous system, that are deeply involved in body metabolism through the release of stress hormones in individuals with PTSD (Aaseth et al., 2019[1]). Indeed, high levels of cortisol, the major stress-related hormone, seems to predispose individuals for altered adipokines (specific cytokines secreted by adipose tissue) accompanied by other metabolic complications that can occur in early life (Michalakis et al., 2013[59]) (Figure 1(Fig. 1)).

Specifically, leptin, adiponectin and ghrelin are adipokines that play a major role in energy homeostasis and appetite regulation. Adiponectin is a signaling hormone made by fat cells and secreted into the bloodstream where it regulates glucose levels by increasing the breakdown of fatty acids. Low adiponectin levels are associated with diabetes, obesity, cardiovascular disease, cancer and PTSD (Gu et al., 2018[22]; Wu et al., 2013[107]). In a recent paper, infusions of adiponectin into the dentate gyrus of the hippocampus in fear-conditioned mice facilitated extinction of contextual fear in this animal model of PTSD (Zhang et al., 2017[109]). A meta-analysis study in 2020 involving 65 articles, showed an inverse link between peripheral adiponectin levels and anxiety, trauma and stress related disorders (Vuong et al., 2020[100]). Furthermore, it has been shown that low levels of adiponectin are connected to an increased probability of PTSD in women (Vuong et al., 2022[99]). 

PTSD also plays a significant role in the regulation of lipid metabolism (von Känel et al., 2010[97]). The severity of PTSD symptoms has been also associated with increased leptin levels and, because of this direct correlation, the authors claimed that leptin may be a valid neuro-endocrinologic marker for the hypervigilant state of vulnerable people who are facing PTSD (Liao et al., 2004[43]). Leptin can also inhibit the transcription of the insulin gene and its secretion (Amitani et al., 2013[5]) and can contribute to the development of oxidative stress via activation of pro-inflammatory cytokines, such as IL-6, IL-2 and tumor necrosis factor (TNF) (Tazawa et al., 2019[88]; Hukshorn et al., 2004[28]). The pro-inflammatory effects of leptin can be associated with a structural and functional similarity to the IL-6 cytokine (Fantuzzi and Faggioni, 2000[19]). 

The role of ghrelin goes far beyond controlling hunger and several studies have shown that peripheral circulating levels of ghrelin are elevated under chronic stress (Lutter et al., 2008[49]). Researchers also found that ghrelin released during chronic stress makes the brain more vulnerable to traumatic events, suggesting that it may predispose people to PTSD (Meyer et al., 2014[58]). A confirmation of these claims came from a paper that showed that elevation of ghrelin may contribute to an increased risk of stress-enhanced fear learning even in adolescents when trauma occurs long after stressor exposure ends, as in PTSD (Yousufzai et al, 2018[108]). In this article, the Yousufzai group analyzed the effects of trauma exposure on the long-term elevation of circulating acyl-ghrelin in Pakistani children who were either injured or lost a loved one in a terror attack (Traumatized group) versus children who had never been injured or lost a loved one in a terror attack (Control group). Only physically healthy children who had no chronic health conditions (including previously diagnosed psychiatric disorders) were included in the study (Yousufzai et al, 2018[108]). The results showed that chronic stress exposure produced an enduring elevation of acyl-ghrelin in the bloodstream of humans exposed to stressors as adolescents. This long-term dysregulation of acyl-ghrelin may, therefore, contribute to a stress-related vulnerability to PTSD that persists long beyond the initial stressor exposure (Hemmann et al., 2012[24]; Lutter et al., 2008[49]; Malik et al., 2020[51]). 

PTSD is also typically associated with an increased risk of type 2 diabetes mellitus (T2DM) (Vancampfort et al., 2016[96]; Rosenbaum et al., 2015[78]; Tanaka et al., 2022[86]). Unhealthy behaviors such as poor eating habits, smoking and substance abuse are often associated with PTSD and might increase the risk for developing diabetes both in men and women. In the first longitudinal study of PTSD and the incidence of T2DM in a civilian sample of women, researchers found that women with the highest number of PTSD symptoms had a nearly 2-fold increased risk of T2DM over follow-up as compared with women with no trauma exposure (Roberts et al., 2015[77]).

PTSD-comorbidity with diabetes was recently reported in a cohort study that examined Veterans Health Affairs medical records from 5916 individuals who were treated for PTSD between 2008 and 2012. After applying eligibility criteria, 1,598 individuals with PTSD and free of diabetes risk were available for analysis. Among veterans who experienced a clinically meaningful reduction in PTSD symptoms over the first year of treatment, 2.6 % developed diabetes during the study, compared with 5.9 % of individuals without that level of improvement in their PTSD (Scherrer et al., 2019[80]).

In war-veteran PTSD individuals it is also very common to see dramatic negative changes in mood and cognitive ability because of traumatic events. A study performed at the VA medical center in Syracuse (New York state), researchers examined the specific health-related problems among people with both PTSD and either depression or diabetes (Trief et al., 2006[90]). They identified 14,795 military veterans with diabetes and looked at whether or not the veterans had PTSD, depression, or other psychiatric diagnoses. The researchers found that compared to control or single co-morbidity groups, people with both PTSD and depression had lower total cholesterol levels and low-density lipoprotein (LDL), higher triglyceride levels, and a higher BMI. In fact, the prevalence of comorbid diabetes and PTSD was 8 % (n = 1129) whereas 57 % of this group (n = 649) also showed comorbidity with major depression symptoms (Trief et al., 2006[90]). Another study that included veterans with PTSD of different ages in Scotland, found a higher risk of diabetes in older veterans especially those with comorbid mood disorder (Bergman et al., 2022[8]). These results give evidence that people with a pre-existing medical condition such as diabetes and who might also have PTSD and depression, may be particularly at risk for metabolic syndrome related health problems.

Several papers have shown that individuals with comorbidity of PTSD and MS also had lower inflammatory biomarkers such as IL-6 and C-reactive protein (CRP) (von Känel at al., 2010[97]), conferring additional health risks to people beyond those related to MS alone (Bastard et al., 2006[7]; Marsland et al., 2010[52]). In particular, in individuals with PTSD that correlates with MS there is a significant increase in production of pro-inflammatory cytokines including TNF-α and IL-6 that can lead to obesity and insulin resistance (Bastard et al., 2006[7]). However, another study showed a decrease in the level of circulating peripheral IL-10 and IL-6 over time while PTSD symptoms still remained (Toft et al., 2022[89]). Altogether, this shows the importance of further research in this field.

## Metabolic Dysfunction in Animal Models of PTSD

To better understand the molecular mechanisms of metabolic dysregulation in humans, several animal models of MS have been developed in recent years. The most common way to recognize these different models as mimicking PTSD-induced trauma is usually to divide them into groups receiving physical, social, or psychological stressors (Borghans and Homberg, 2015[11]). Physical stressors such as restraint stress, foot shock stress, or enhanced fear learning are usually associated with the use of aversive stimuli to stress the subjects and compare these to traumatic near-death experiences like those experienced by soldiers at war. Social stressors relate more to models that expose vulnerable people susceptible to PTSD to common and repetitive traumas, such as lack of social interaction, house or social instability, or early life stress. Finally, psychological stressors, unless physical and social, rely mostly on the study of human vulnerability to trauma and their susceptibility or resilience to PTSD, that can translate in the animal model as the instinctive response to a natural predator. 

For example, several authors used a predator scent stress (PSS) to confront animal subjects with the scent of their natural predators. For instance, in a paper published by Tsikunov and coauthors demonstrated decreased concentration of HDL and triglycerides in rat serum after psychic trauma (Tsikunov et al., 2006[91]). Moreover, rats that were exposed to repeated snake aggression showed a decrease in HDL cholesterol levels over a span of 6 weeks and sharp increases in serum triglycerides mimicking the same outcome as results for individuals with an increased risk for cardiovascular disease (Tsikunov et al., 2006[91]). These data suggest that physic trauma is accompanied by permanent changes in lipid metabolism. Changes in the analyses of differentially expressed genes associated with MS were also observed in blood, brain, and spleen of 8-10-weeks-old C57BL/6 male mice (Muhie et al., 2015[66]). Mice C57BL/6 were exposed to intruder SJL male 6 hours/day for 5 or 10 days and activations of behavioral responses associated with anxiety disorders was observed (Table 1(Tab. 1); References in Table 1: Bersani et al., 2016[9]; Gautam et al., 2015[21]; Jones et al., 2015[31]; Karlović et al., 2004[34]; Koirala et al., 2023[37]; Levkowitz et al., 2015[41]; Liu et al., 2016[46]; Mellon et al., 2019[56]; Miller et al., 2014[62]; Ogłodek et al., 2015[69]; Sawicki et al., 2015[79]; Tsikunov et al., 2006[91]; von Känel et al., 2010[97]; Wohleb et al., 2014[105]) (Muhie et al., 2015[66]). Differentially regulated genes found in the study of Muhie and colleagues (2015[66]) were significantly associated with signaling pathways implicated in PTSD comorbidities associated with metabolism including insulin signaling pathway, mTOR signaling, Type I and II diabetes mellitus (Muhie et al., 2015[66]). Metabolites levels in plasma were tested in aggressor-exposed male mice (C57BL/6J) (Gautam et al., 2015[21]). Mice were stressed by exposures to trained aggressor mice albino SJL for 5 or 10 6-hour sessions daily and showed plasma metabolite alterations on 24 hours or even 1.5 or 4 weeks after the last stress session (Gautam et al., 2015[21]). Higher numbers of altered metabolites were observed at 24 hours after the last stress session as compared to after 1.5 or 4 weeks of stress-withdrawal. Moreover, the major metabolic fuels, carbohydrates, amino acids, and lipids were higher in plasma of aggressor-exposed male mice (Gautam et al., 2015[21]). 

Despite the lack of PTSD variability in animal models compared to humans, overall, they can help us understand metabolic changes developed by chronic stress and insufficient coping mechanisms. For example, a recent study on mice investigated effects of chronic variable stress on metabolism in skeletal muscle. They found shifts in the respiratory exchange ratio from carbohydrates to fatty acid oxidation and a change in the mitochondrial proteome (Nikolic et al., 2023[67]). The limitations of validity must be considered before taking into account investigation of PTSD on animal models. To induce PTSD in humans it must be exposed to a life-threating event. Life-threatening stressors including predator exposure and resident-intruder have a construct validity in an animal model (Gautam et al., 2015[21]; Muhie et al., 2015[66]).

**Figure 2:**
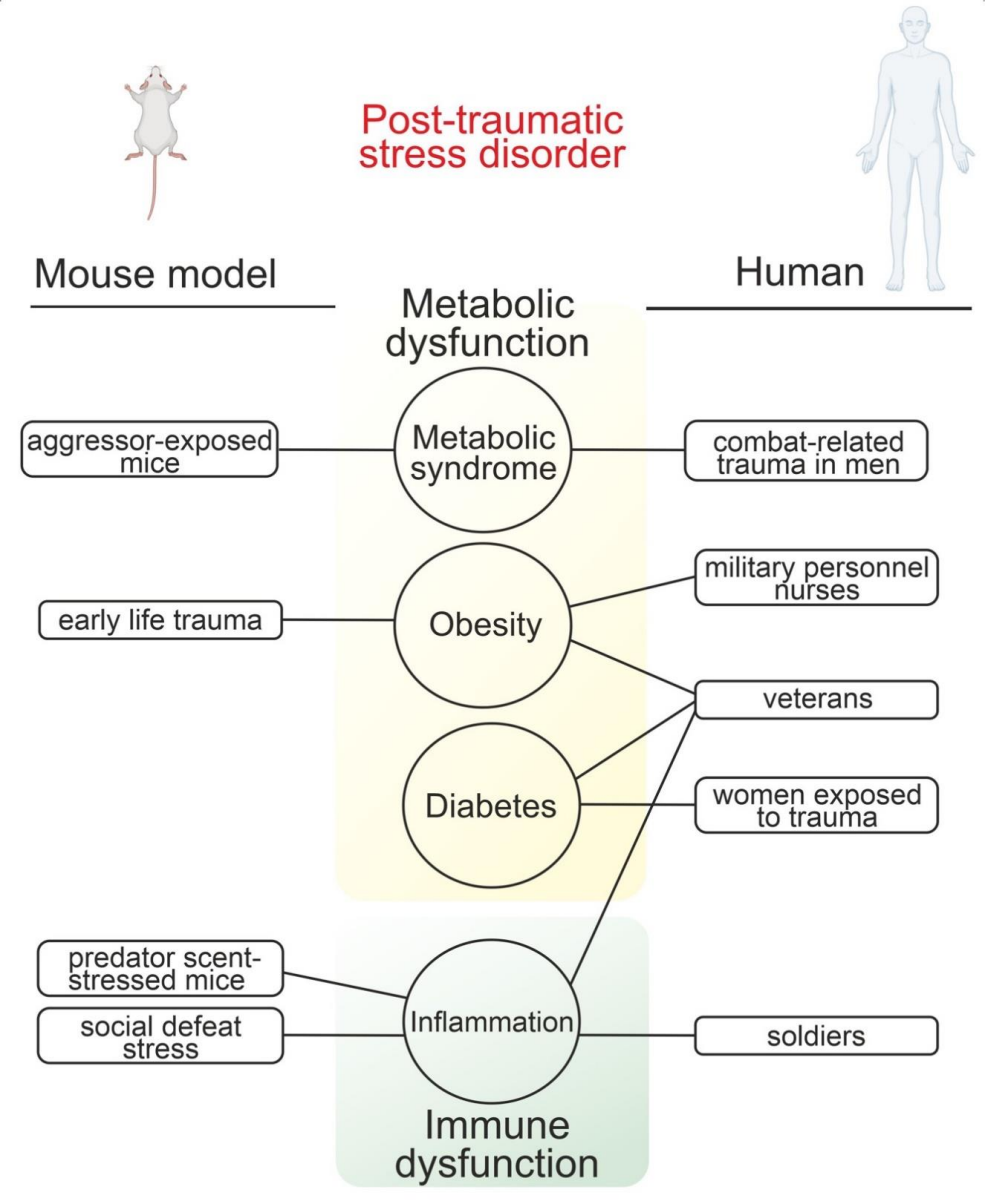
Metabolic and immune responses in rodents and human. Physical or psychosocial stressors used to generate animal models of post-traumatic stress disorder that is associated with metabolic syndrome, obesity, diabetes and inflammation.

Pre-existing metabolic diseases contribute greatly in the development of PTSD. Indeed, rats with preexisting diabetes had more pronounced PTSD-like symptoms (Ribeiro et al., 2021[76]). While these rodent models result in some PTSD-like phenotypes consistent with the clinical data and suggest that MS predisposes individuals to PTSD (Mellon et al. 2018[57]) (Figure 2(Fig. 2)), the mechanisms of this correlation between metabolic dysregulations and PTSD still remain unknown (Johannessen et al., 2013[30]).

## PTSD Induced Immune Dysfunctions in Humans

The immune inflammatory response is recognized as a key element in the pathogenesis of PTSD (Kim et al., 2020[35]). The main system responsible for activation of the immune response under trauma was shown to be the multiple intricate interconnected pathways between the hypothalamic-pituitary-adrenal (HPA) axis and the adrenergic nervous system (Passos et al., 2015[71]; Kinlein et al., 2015[36]). In a normal condition of a mild stressor event the HPA axis and the sympathetic nervous system (SNS) react quickly to an acute challenge by (a) releasing glucocorticoids into the bloodstream, (b) compensating cardiovascular and hemodynamic dysfunctions, and (c) increasing awareness and increasing energy mobilization to face the changes (Molina, 2005[65]; Kinlein et al., 2015[36]).

During an acute or chronic stressful event glucocorticoids released by the adrenal cortex, that are the effector hormones of HPA axis, are fundamental for modulating and regulating the immune response (Kinlein et al., 2015[36]). In fact, glucocorticoids work as an immune suppressor under stress and their bloodstream fluctuations have been shown to correlate with decreases in IL-6 and TNF-α level (Deslauriers et al., 2017[13]; Hori and Kim, 2019[26]). Interleukin-1β (IL-1β) is another small cell-signaling protein in the brain that has a regulatory role in the immune system, is also modulated by glucocorticoids, and has been found to be elevated in cases of PTSD (Jones et al., 2015[31]; Oglodek, 2022[68]).

Under pathological conditions, such as those present in PTSD individuals, a prolonged duration and activation of stress can trigger deleterious effects on immune functions (Wang et al., 2017[101]). Other studies have shown that in individuals with PTSD, the levels of pro-inflammatory cytokines, such as IL-6 (Maes et al., 1999[50]), IL-1β (Jones et al., 2015[31]), or IL-2 (Smith et al., 2011[82]) are augmented, and these levels are positively correlated with PTSD symptoms in traumatized individuals (von Känel et al., 2007[98]). Also, along with pro-inflammatory cytokines, levels of the complement protein CRP (Spitzer et al., 2010[83]) are consistently increased in PTSD individuals as compared to war veterans versus controls (Michopoulos and Jovanovic, 2015[60]). These results were further confirmed by a systematic meta-analysis that showed that increased inflammation is coincident with PTSD in traumatized individuals (Passos et al., 2015[71]). Recently, a correlation between the concentration of proinflammatory cytokines and the severity of depression symptoms has also been demonstrated (Ogłodek, 2022[68]). Taken together, these results show that inflammation is clearly present during PTSD symptomatologic conditions. Moreover, immune factors might not only be markers for this symptom state, but also contribute to a pre-existing risk for PTSD upon trauma exposure (Plantinga et al., 2013[73]). Indeed, several papers have shown a link between a pre-existing risk for PTSD among military service members before a combat trauma actually happened (Eraly et al., 2014[18]). To confirm these data, altered gene expression in peripheral immune cells collected from soldiers who developed PTSD before combat were found to be similar to those of soldiers that did not go into combat but eventually developed PTSD afterwards. Researchers found that a functional mutation in genes related to the immune system was indeed associated with an increased risk for PTSD (van Zuiden et al., 2012[95]). Overall, these findings support the theory that alterations in the immune system may not only be the consequence of a trauma but also promote the development of PTSD symptoms. However, the causal link between symptoms and inflammation remains to be understood.

To better study the fundamental inflammatory processes involved in PTSD, several widely accepted animal models have been used over the last two decades (Zoladz and Diamond, 2016[111]; Deslauriers et al., 2018[14]). Many publications have used animals as valuable contributors to learn more about the disorder and its outcomes. However, not all animal models are suitable to answer all questions regarding the diverse aspects of PTSD (Wang and Young, 2016[102]). For example, to study immune responses triggered by neuroinflammation in the CNS, a common method is to induce peripheral inflammation by injecting rodents with endotoxin (Cazareth et al., 2014[12]). Immediately after this insult, immune mediators such as IL-1, IL-6, and TNF-α, can cross the blood-brain barrier and activate the immune response in the CNS (Pan et al., 2011[70]). Several papers have shown that inflammatory processes disrupt hippocampal function induced by pro-inflammatory cytokines with a concomitant increase in the number of activated microglia induced by peripheric inflammatory activation (Williamson and Bilbo, 2013[103]). However, with the presence of over 20 independent PTSD reported symptoms, to find the right animal model for replicating all aspects of the disorder can be challenging and unrealistic.

## Immune Dysfunctions in Animal Models of PTSD

Several animal models for PTSD studies have been established (Deslauriers et al., 2018[14]). Besides the use of inescapable shock, such as foot tail-shock, that is considered to be one of the most adverse stressors (although not etiologically valid), in rodent models of fear, another commonly used model involves repeated exposure to either the rodent's natural predator or urine of the predator (usually cats or foxes). Several papers have shown that the stressful effects of these behavioral paradigms were still present up to 3 months after stress exposure resulting in hyperarousal, exaggerated fear response, and general avoidance, among others (Zoladz and Diamond, 2016[111]; Deslauriers et al., 2017[13]). 

Similar to what has been shown regarding the dysregulation of cytokines seen peripherally in human subjects with PTSD, predator stress was able to induce long-term inflammation in the brain resulting in an increased level of pro-inflammatory cytokines, whereas the levels of the anti-inflammatory ones were reduced (von Känel et al., 2007[98]; Wilson et al., 2014[104]).

Constructs of animal models of fear conditioning are also highly relevant to study the immunological effects induced by severe stressors in PTSD (Deslauriers et al., 2017[13]). Indeed, in a fear-learning model Jones and coworkers (2015[31]) showed that hippocampal IL-1 immunoreactivity levels were increased because of the severe stressor and that these effects were prevented by using an IL-1 receptor antagonist within 24-48 hours after the stress. Increase in hippocampal IL-1β in male Sprague Dawley rats that were exposed to foot shock (2 mA, 1 s) during 90 min on a 6-min variable time schedule is associated with stress-enhanced fear learning (Jones et al., 2015[31]). Therefore, these results suggest that IL-1 may play a causal role in PTSD following trauma exposure, after the stress has been triggered by the fear learning response (Jones et al., 2015[31]). Moreover, site-specific injections of both TNFα and IL-6 into the amygdala have been shown to impair the acquisition and extinction of fear conditioning (Hao et al., 2014[23]). Taken together, these data suggest that increased inflammation in the CNS in a fear conditioning model of PTSD may serve as the biological trigger for metabolic alterations in individuals with PTSD. 

In animal models of PTSD such as in predator scent-stressed mice, anxiety could be induced by activation of the pro-inflammatory NF-κB pathway. Interruption of this stress-provoked pro-inflammatory NF-κB pathway reduced the elevated IL-1 levels and, as a consequence, mitigated anxiety levels in the predator-stressed mice (Zimmermann et al., 2012[110]). Psychogenic stress induced by predator scent stress (PSS) leads to cytokines' activation in rodent model (Levkowitz et al., 2015[41]). Placing male Sprague-Dawley rats on well-soiled cat litter for 10 min caused significant increase in IL-6 and TNF-α levels in the brain regions (Levkowitz et al., 2015[41]). These data demonstrated proinflammatory response associated with PTSD (Levkowitz et al., 2015[41]) and are also confirmed in male combat veterans from the Iraq (Bersani et al., 2016[9]).

Single prolonged stress (SPS) is often used to model key symptoms of PTSD (Laukova et al., 2014[39]). It was demonstrated enhanced oxidative stress and neuroinflammation in the hippocampus after SPS (Liu et al., 2016[46]). Sprague-Dawley rats were restrained for 2 h followed by forced swimming and diethyl ether to induce PTSD phenotype (Liu et al., 2016[46]). Significant increase in IL-6 levels at day 7 after SPS was observed (Liu et al., 2016[46]).

Social defeat stress has also been used to assess immune responses related to prolonged and repeated stress exposure (Deslauriers et al., 2017[13], 2018[14]). In mice that were exposed to repeated social defeat during the resident intruder test, several PTSD-relevant stress-related biological and molecular phenotypes were discovered, and social defeat was found to induce both peripheral and central inflammation. The immune system may play different roles depending on the chosen social defeat paradigm. There are types of social defeat stress that involve injuries to mice that can lead to the participation of peripheral immune cells in the final outcome (Stein et al., 2017[84]). These stress paradigms were shown to induce activation of the HPA axis and to stimulate the trafficking of CG-insensitive monocytes to tissues including the spleen, lung, and brain (Reader et al., 2015[75]). Moreover, repeated social defeat increased mRNA expression of IL-1β, CCL2, and CXCL2 in microglia/macrophages but not in astrocytes, indicating that key pro-inflammatory mediators were elevated in the brain (Sawicki et al., 2015[79]). Sawicki and colleagues suggested translational relevance of their data because psychosocial stress in humans promotes a pro-inflammatory state within the CNS (Iwata et al., 2013[29]). Higher levels of pro-inflammatory mediators in the CNS contribute to the development of anxiety and depressive-like symptoms. Furthermore, if mice were splenectomized prior to the repeated social defeat, the monocyte trafficking and anxiety behavior that otherwise occurs following subthreshold stress were prevented (McKim et al., 2018[55]). Finally, studies of pharmacological treatments in animal models of pathological stress that target inflammatory mechanisms may be effective treatment strategies for PTSD since inflammation is a fundamental part of the etiology and maintenance of this disease (Michopoulos and Jovanovic, 2015[60]). 

Among several different animal models of PTSD, social defeat studies with a predator exposure model were conducted to better understand the results of selective serotonin reuptake inhibitors (SSRIs) on the impact of inflammatory mediators in human subjects (Amitai et al., 2016[4]). Sertraline, one of the most commonly used SSRIs, was shown to influence inflammation and neurotransmitter modulation in rodent model (Wilson et al., 2014[104]). In the study of Wilson and colleagues male Sprague-Dawley rats (n = 6/group × 4 groups) were placed in Plexiglas cylinders to a cage with a cat for 1 h on days 1 and 11 of a 31-day stress regimen to induce predator exposure/psychosocial stress (Wilson et al., 2014[104]). The data showed reduced levels of the pro-inflammatory mediator IL-1β and signaling of the pro-inflammatory receptor TLR4 in the hippocampus and prefrontal cortex (PFC), while increasing the levels of anti-inflammatory cytokines IL-4 and IL-10 in the same areas (Wilson et al., 2014[104]). Sertraline was shown to have anti-inflammatory effects, however, provided no positive effects on anxiety or behavior (Wilson et al., 2014[104]).

## Conclusions and Perspectives

The animal models of trauma-related inflammation for PTSD addressed in this review provide a rich source of evidence that confirms the involvement of systemic inflammation and metabolic dysfunction in the pathogenesis of PTSD and provide new translational evidence. Tightly regulated interactions between nervous and immune systems and metabolism (Mellon et al., 2018[57]) are linking stress to inflammation and metabolic syndrome, the latter two conditions being also associated with cancer (Levine et al., 2014[40]) and other chronic diseases (Blessing et al., 2017[10]). It is well established that low-grade inflammation causes, or is at least associated with, metabolic deteriorations. With some limitations, we can talk about dysregulation of neuro-immuno-endocrinological mechanisms of homeostasis and/or redox metabolism in PTSD individuals (Rosenbaum et al., 2015[78]). A deeper understanding of early derangements in these dysregulations and determination of the context of changes within the regulatory triangle (nervous-endocrine-immune systems) will unlock new information for more accurate targeting of relevant pathophysiological mechanisms (Serhiyenko et al., 2022[81]). This will allow (a) development of interventions to correct existing problems, and (b) identify relevant biomarkers to control efficacy and, if needed, to modify treatment as was clinically demonstrated for interleukin-6 (IL-6) as immunological biomarker (Michopoulos et al., 2016[61]). Therefore, dissecting these psycho-somatic interactions and identifying early changes in individuals is expected to be key not only in developing more accurate diagnostics but also in the search for interventions capable of arresting PTSD progression and preventing the development of long-term complications. PTSD has been linked to elevated risks of chronic health conditions. Moreover, PTSD appears to be less prevalent in older than younger adults. Indeed, accumulating evidence has linked PTSD to multiple aging-related chronic health conditions. Being a co-morbidity to many diseases, aging and related functional declines, PTSD can be treated pharmacologically (Piskovatska et al., 2019[72]; Lushchak et al., 2020[48]; Vaiserman et al., 2020[92][94], 2021[93]). Several pharmacological agents including resveratrol, rapamycin, metformin and aspirin show the effectiveness for reduction inflammation, prevention CVD, and slow down the functional decline in certain organs (Lushchak et al., 2020[48]; 2023[47]; Piskovatska et al., 2019[72]; Vaiserman et al., 2020[92][94]).

Better understanding of cause-consequence relationships between psycho-emotional stress, endocrine/metabolic and immune dysfunctions is urgently needed in order to identify key mechanisms responsible for the progression of this disease. In turn, this will enable possibilities of targeted interventions that will be capable of intercepting PTSD progression early enough to prevent detrimental complications. It may well be that a certain degree of heterogeneity of the disease may require different therapeutic approaches for specific sub-types of PTSD. It becomes clear that complex holistic approaches than will assess the degree of involvement and damage of related regulatory mechanisms are required to adequately address the diversity of clinical manifestations and pathological processes taking place in individuals with PTSD.

## Declaration

### Acknowledgments

This work was partially supported by the grant from Ministry of Education and Science of Ukraine [grant number 0123U101790].

### Conflict of interest

There are no conflicts of interest to declare.

## Figures and Tables

**Table 1:**
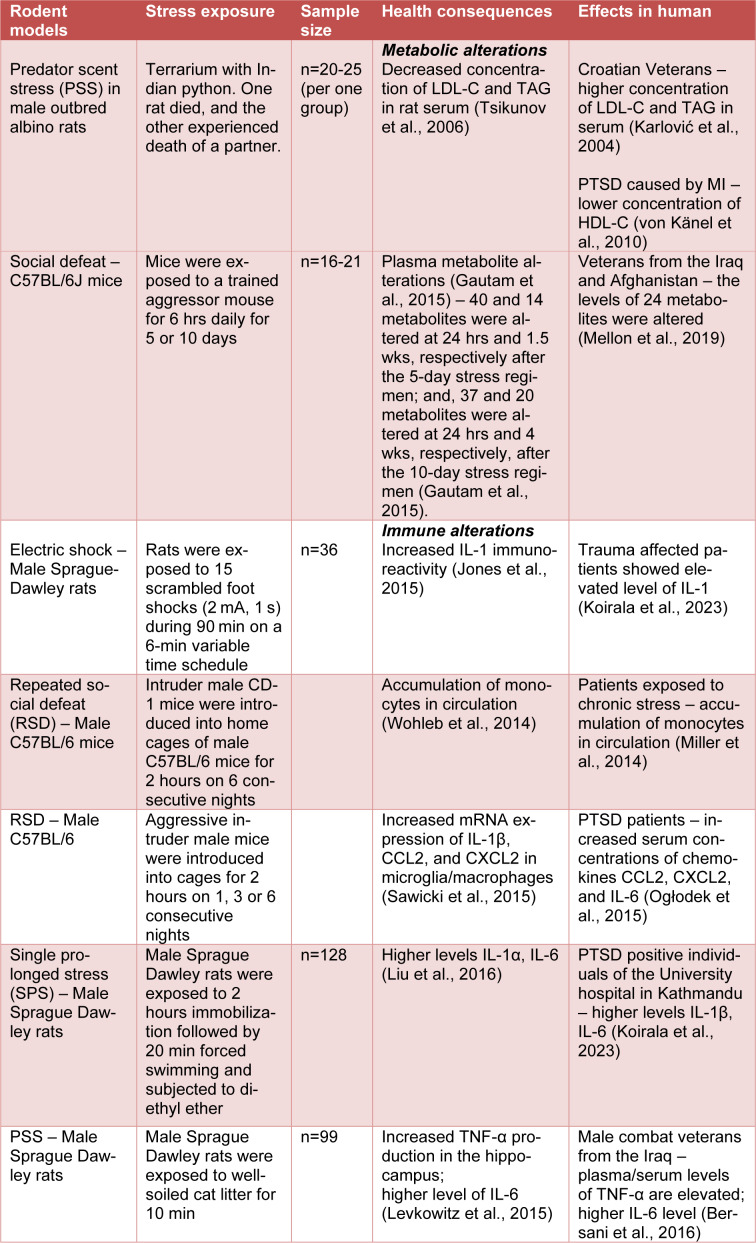
Description of rodent models of PTSD with metabolic and immune consequences
